# Right haemothorax secondary to pulmonary vein laceration following left-sided pacemaker implantation: a case report

**DOI:** 10.1093/ehjcr/ytaf093

**Published:** 2025-02-25

**Authors:** Simon Fitouchi, Loïc Faucher, Aissam Labani, Laurence Jesel, Halim Marzak

**Affiliations:** Department of Cardiovascular Medicine, Nouvel Hôpital Civil, University Hospital of Strasbourg, 1 place de l'Hopital, Strasbourg 67000, France; Department of Cardiovascular Medicine, Nouvel Hôpital Civil, University Hospital of Strasbourg, 1 place de l'Hopital, Strasbourg 67000, France; Department of Radiology, Nouvel Hôpital Civil, University Hospital of Strasbourg, 1 place de l'Hopital, Strasbourg 67000, France; Department of Cardiovascular Medicine, Nouvel Hôpital Civil, University Hospital of Strasbourg, 1 place de l'Hopital, Strasbourg 67000, France; UR 3074 Translational CardioVascular Medicine CRBS, 1 rue Eugène Boeckel, Strasbourg 67000, France; Department of Cardiovascular Medicine, Nouvel Hôpital Civil, University Hospital of Strasbourg, 1 place de l'Hopital, Strasbourg 67000, France

**Keywords:** Pacemaker, Atrial lead perforation, Haemothorax, Complication, Case report

## Abstract

**Background:**

Lead perforation is a rare but potentially fatal complication of cardiac device implantation. Atrial lead perforations, though less common than ventricular ones, can have severe consequences, especially in elderly patients with underlying structural heart disease.

**Case summary:**

An 83-year-old male with amyloid and valvular cardiomyopathy underwent dual-chamber pacemaker implantation for third-degree atrioventricular block following transcatheter aortic valve implantation. The active-fixation atrial lead was positioned on the right atrial free wall. Six hours post-implantation, the patient developed haemodynamic instability. Echocardiography revealed a small pericardial effusion, while pacemaker interrogation showed an increased atrial pacing threshold. Chest imaging demonstrated a moderate right haemothorax. Initially, conservative management was recommended. However, the patient’s condition deteriorated, progressing to cardiac arrest requiring brief cardiopulmonary resuscitation. Emergency sternotomy revealed a large right haemothorax and laceration of the right upper pulmonary vein in contact with the atrial lead screw. The laceration was repaired, and the atrial lead was repositioned. The patient recovered well and was discharged 5 days after surgery.

**Discussion:**

Lead perforation is an uncommon but potentially severe complication of pacemaker implantation, with an incidence of 0.5–1% of cases. Several risk factors have been identified, including advanced age, low body mass index, atrial remodelling, and lateral lead placement. In this case, the atrial lead perforation extended from the atrial wall to the right superior pulmonary vein. This case underscores the potential risks associated with lateral lead positioning in geriatric patients and emphasizes the necessity for meticulous lead placement and vigilant post-implantation monitoring to mitigate complications.

**Learning points**

- Atrial lead perforation can cause severe complications like haemothorax without tamponade, potentially injuring structures such as the right superior pulmonary vein.
- Computed tomography imaging may fail to detect low-volume intrathoracic bleeding, especially in case of artefacts related to pacemaker lead. Clinical assessment remains crucial for diagnosing complications despite reassuring initial scans.
- Risk factors for lead perforation include advanced age, low body mass index, atrial remodelling, and placement on lateral walls. Meticulous evaluation and optimal lead positioning are crucial to reduce the risk of perforation during device implantation.

## Introduction

Cardiac perforation during pacemaker implantation is a rare but potentially life-threatening complication. We present a case of right superior pulmonary vein (RSPV) laceration caused by an active-fixation atrial lead, resulting in a contralateral haemothorax in an octogenarian patient following dual-chamber pacemaker implantation after transcatheter aortic valve implantation (TAVI). This unusual complication underscores the critical importance of meticulous post-procedural monitoring and prompt multidisciplinary intervention in the event of clinical deterioration.

## Summary figure

**Figure:**
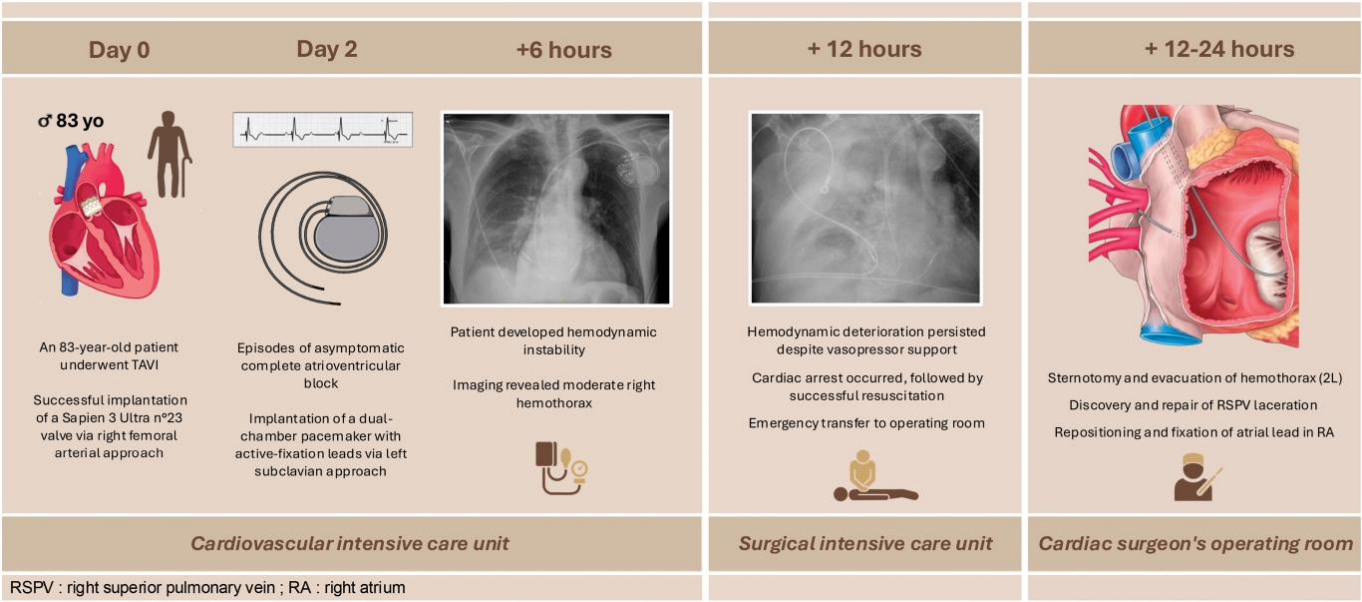


## Case summary

An 83-year-old male patient was admitted to our cardiology department for TAVI. The patient had a history of amyloid and valvular cardiomyopathy with preserved left ventricular ejection fraction and severe calcific aortic stenosis (peak gradient 55 mmHg, mean gradient 41 mmHg, and valve area 0.65 cm^2^), with predominantly septal left ventricular hypertrophy and no obstruction. The patient was on apixaban for paroxysmal atrial fibrillation. He was 170 cm tall and weighed 56 kg [body mass index (BMI) 19.38 kg/m^2^). The 12-lead resting electrocardiogram (ECG) on admission showed sinus rhythm with first-degree atrioventricular block (PR interval 470 ms), complete right bundle branch block (QRS duration 172 ms), and left anterior fascicular block with an axis of −153° (see Supplementary material online, *Figure S1*).

A Sapien 3 Ultra 23 mm valve was successfully implanted via the right femoral artery under local anaesthesia. On post-operative Day 2, telemetry monitoring revealed brief episodes of asymptomatic third-degree atrioventricular block with a ventricular rate of 41 b.p.m, necessitating implantation of a dual-chamber pacemaker.

**Figure 1:**
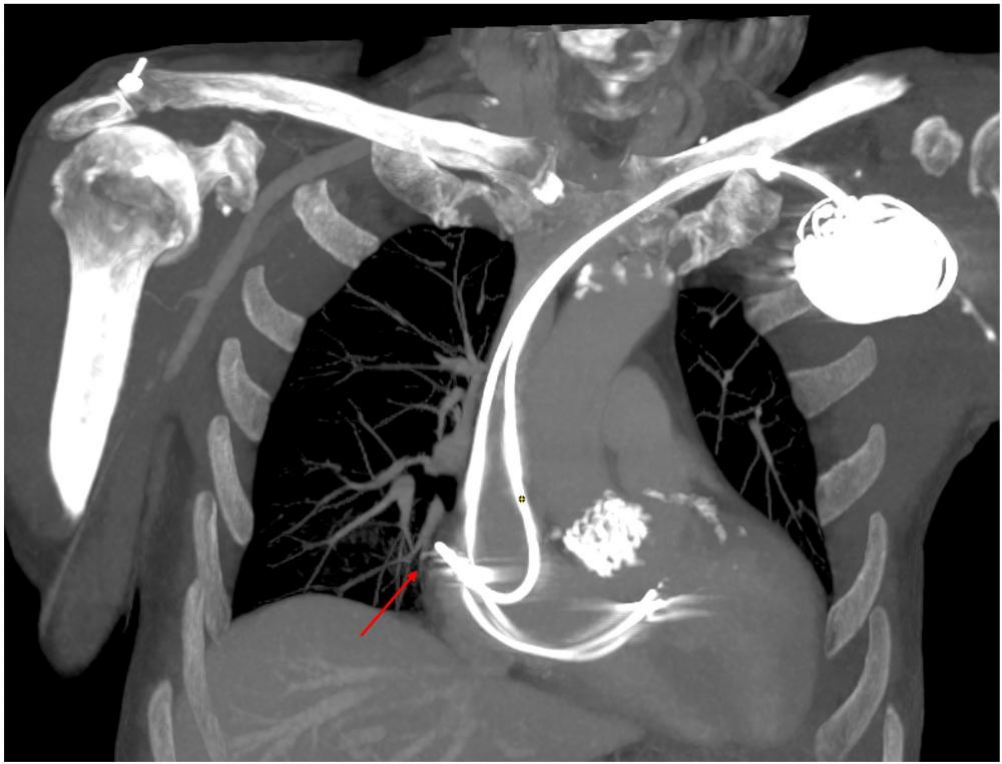
Chest computed tomography scan 6 h post-implantation revealing perforation of the atrial lead through the right atrial wall, in contact with the right pulmonary venous tree, without contrast extravasation (arrow).

A dual-chamber pacemaker (Medtronic Azure XT DR) was implanted under local anaesthesia on the left side. Due to the absence of cephalic access, active-fixation pacemaker leads were introduced via the left subclavian vein. The ventricular lead (Medtronic 4076–58 cm) was screwed into the mid-septum with 18 turns of the screw using a RVOT Stylet Kit (St. Jude Medical). The atrial lead (Medtronic 4076−52 cm) was positioned multiple times in the right atrial appendage using an appropriate J-curved stylet. However, atrial pacing thresholds were excessively high (3 V/1 ms), likely due to atrial remodelling. The atrial lead was ultimately fixed with 17 turns of the screw, in accordance with the manufacturer’s recommendations, to the posterolateral region of the free wall of the right atrium (RA) (*Figure 1*). This final position achieved adequate atrial sensing (1 mV) and a pacing threshold of 0.75 V at 0.4 ms at the time of implantation.

Upon return from the operating room, the patient was asymptomatic. Physical examination was unremarkable. The ECG demonstrated atrial and ventricular pacing at 60 b.p.m. with adequate capture (see Supplementary material online, *Figure S2*). Follow-up echocardiography revealed satisfactory function of the aortic bioprosthesis and no evidence of pericardial effusion.

**Figure 2:**
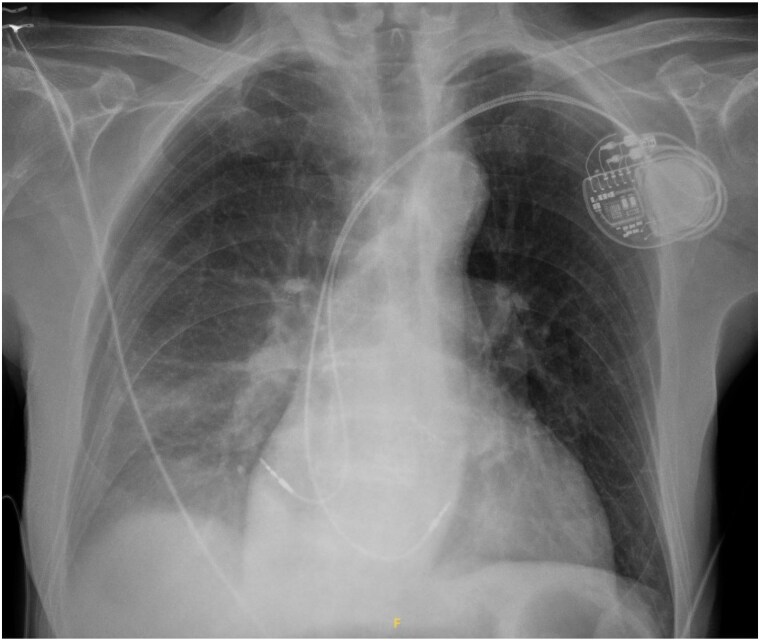
Chest radiograph obtained 6 h after pacemaker implantation demonstrating a large right-sided pleural effusion extending to the mid-lung field.

At H + 6 post-procedure, the patient became haemodynamically unstable. He was eupnoeic with a heart rate of 60 b.p.m. in sinus rhythm and hypotensive with a blood pressure of 93/47 mmHg (mean arterial pressure 52 mmHg). Pulmonary auscultation demonstrated asymmetry with decreased breath sounds on the right side. There were no signs of shock, such as mottling or cold extremities. Follow-up echocardiography revealed a 5 mm left ventricular lateral pericardial effusion (see Supplementary material online, *Figure S3*). Pacemaker interrogation revealed an elevated atrial pacing threshold of 3 V/0.4 ms in bipolar configuration and complete absence of atrial capture at 5 V/1 ms in unipolar configuration, suggestive of atrial lead tip perforation. Chest radiography (*Figure 2*) revealed an opacity extending to the mid-lung field without evident obliteration of the diaphragmatic dome or costophrenic angles. No mediastinal shift was observed. The cardiac device leads appeared to be in appropriate position. The atrial lead was in contact with the RA free wall. Chest computed tomography (CT) demonstrated a moderate unilateral right haemothorax without visible contrast extravasation in arterial or venous phases. The tip of the atrial lead was in contact with the lateral wall of the RA, without evidence of parietal breach or indirect signs of rupture. A small pericardial effusion was observed, with integrity of the large mediastinal vessels (*Figure 1*; Supplementary material online, *Figures S4* and *S5*).

After consultation with cardiac surgeons and considering the patient’s haemodynamic stability, we decided against emergent lead repositioning or effusion drainage due to the patient’s anticoagulation with apixaban. Laboratory tests revealed anaemia (haemoglobin 11.2 g/dL, *n* > 13 g/dL) and elevated high-sensitivity troponin T (241 ng/L, *n* < 14 ng/L). The patient was transferred to the surgical intensive care unit for close monitoring. To reverse the effects of apixaban, 500 IU of prothrombin complex concentrate was administered.

**Figure 3:**
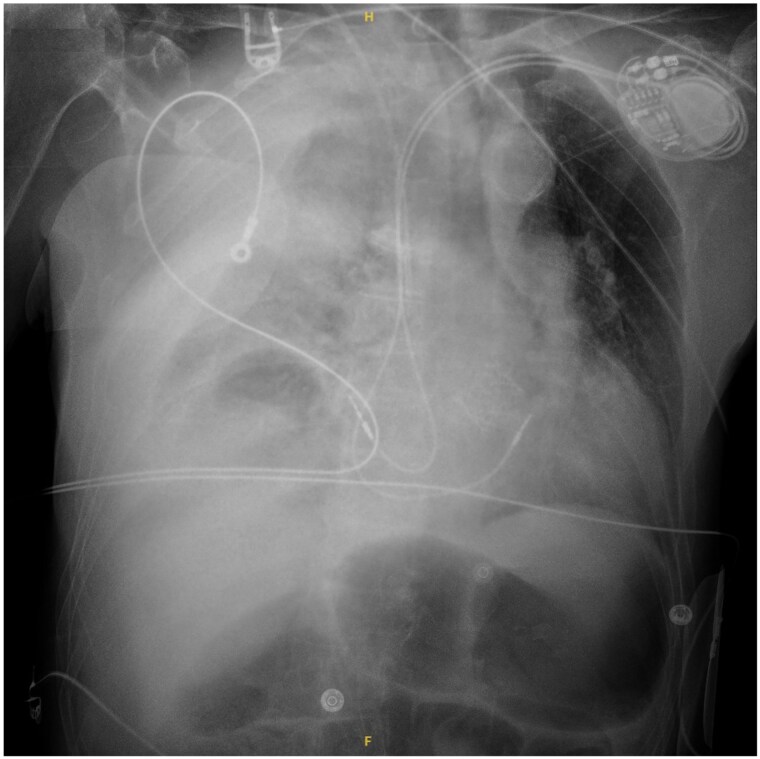
Chest radiograph obtained 12 h after pacemaker implantation showing significant worsening of the effusion with homogeneous opacification of the right hemithorax.

At H + 12 post-procedure, the patient’s haemodynamic status deteriorated despite initiation of vasopressor support with norepinephrine. He experienced cardiac arrest with an electro-mechanical dissociation. Immediate cardiopulmonary resuscitation was initiated. The patient regained spontaneous circulation after 2 min of chest compressions and administration of 1.5 mg of epinephrine (no-flow time 0 min and low-flow time 2 min). Transthoracic echocardiography excluded cardiac tamponade. Pleural ultrasound demonstrated worsening of the right pleural effusion without evidence of pneumothorax. Chest radiography revealed complete opacification of the right hemithorax (*Figure 3*). Arterial blood gas analysis indicated severe anaemia with haemoglobin of 7.2 g/dL (*n* > 13 g/dL), haematocrit 22.1% (*n* > 35%), and hyperlactataemia of 4 mmol/L (*n* < 1.6 mmol/L).

The patient was urgently transferred to the operating room for emergency surgical intervention. A vertical median sternotomy was performed to access the thoracic cavity. Upon opening the right pleura, a large 2-L haematoma was evacuated. Inspection revealed a laceration of the RSPV in contact with the screw-in helix of the atrial lead. The laceration was repaired using 5-0 Prolene non-absorbable suture. A small amount of clotted blood was visualized in the pericardial space. Examination of the RA revealed a pseudoaneurysm caused by the atrial lead. Subsequently, the atrial lead was secured within the RA.

The patient was rapidly extubated and transferred to the cardiac intensive care unit. His clinical course was favourable. Pacemaker interrogation demonstrated satisfactory electrical parameters for both atrial and ventricular leads. Follow-up echocardiography revealed no pericardial effusion, and chest radiography showed no recurrence of haemothorax (see Supplementary material online, *Figure S6*). The patient was discharged on post-operative Day 5 (7 days after initial admission).

## Discussion

Lead perforation is a rare but potentially severe complication, occurring in approximately 0.5–1% of pacemaker implantations.^1^ Most cases occur during implantation or within the first 24 h post-procedure, as in our case. Delayed lead perforation can sometimes appear several days or even months after the procedure.^2^ Some authors report that atrial leads perforate more frequently than ventricular leads, and ventricular implantable cardioverter-defibrillator leads perforate more frequently than ventricular pacemaker leads.^3,4^ Several factors may contribute to lead perforation: overscrewing of the lead, inadequate positioning in high-risk areas (lateral wall of the RA^5^ and right ventricular apex^6,7^), fragility of the myocardial wall (recent infarction and corticosteroid therapy^8^), and use of thin and rigid leads.

Our patient presented several risk factors: advanced age with low BMI,^5^ atrial remodelling associated with amyloid and valvular heart disease, and positioning of the atrial lead on the lateral wall of the RA. The difficulty encountered in achieving adequate atrial lead placement highlights the anatomical challenges that can arise during pacemaker implantation, particularly in elderly patients with structural heart disease. The decision to screw the atrial lead on the lateral wall of the RA, while not ideal, was a reasonable compromise given the circumstances described above. A significant current of injury on the atrial intracardiac electrogram was systematically assessed after atrial lead implantation in the operating room. However, we did not routinely perform unipolar pacing threshold tests when bipolar pacing thresholds were adequate and a satisfactory current of injury was present. Conducting these tests would have provided additional and more precise information regarding the position of the atrial lead and the quality of its contact with the cardiac tissue. The placement of the atrial lead in the right high-septal area near Bachmann’s bundle would be a safe and feasible alternative for our high-risk patient. This atrial pacing site is associated with a lower incidence of atrial arrhythmias compared to other atrial pacing locations.^9^ However, it is important to note that there is a slightly higher risk of lead dislocation, particularly in patients with a dilated RA.

However, this case demonstrates that such compromises may lead to unforeseen complications.

**Figure 4:**
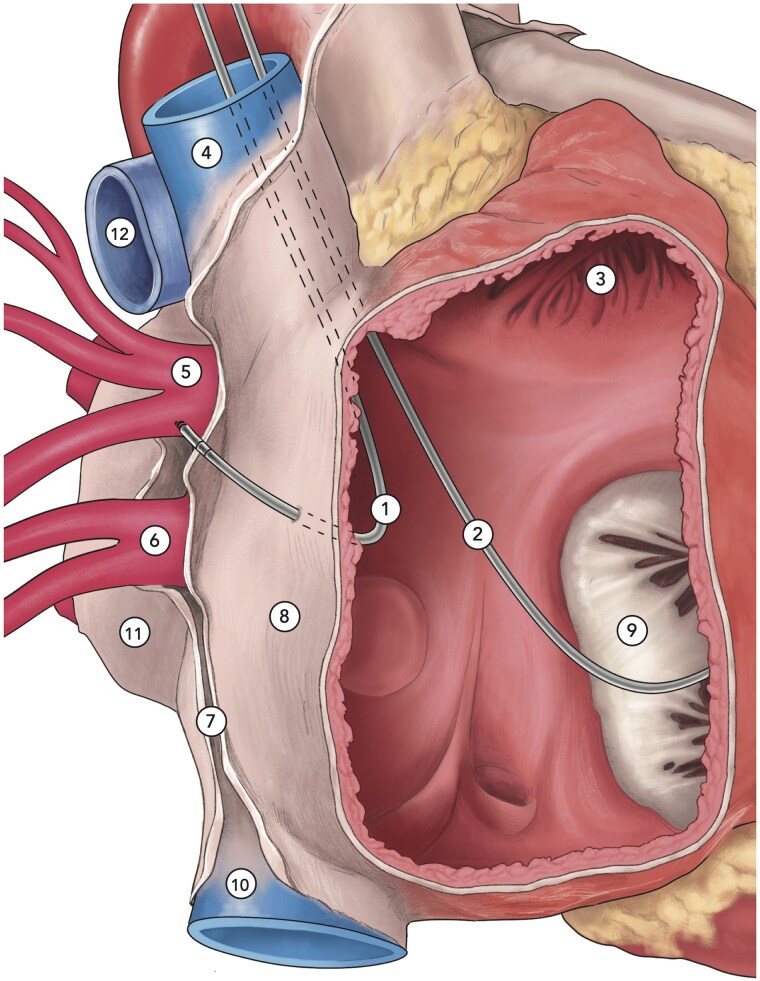
Anatomical diagram of the right lateral view of the opened right atrium, illustrating the atrial lead perforating the posterolateral region of the atrial free wall and the pericardium extending to the right superior pulmonary vein. 1, atrial lead; 2, ventricular lead; 3, right atrial appendage; 4, superior vena cava; 5, right superior pulmonary vein; 6, right inferior pulmonary vein; 7, pericardium; 8, posterolateral wall of the right atrium; 9, tricuspid valve; 10, inferior vena cava; 11, left atrium; 12, right pulmonary artery.

Several cases of cardiac tamponade or pneumothorax^10^ related to atrial lead perforation have been frequently described in the literature. Haemothorax is an extremely rare complication of pacemaker implantation. Only one case^5^ related to atrial lead perforation has been reported to date. In our case, we also observed a contralateral haemothorax without tamponade resulting from an injury to the RSPV caused by atrial lead perforation. The risk of perforation is particularly concerning in this location due to the thinness of the atrial wall.^11^ The pathophysiological mechanism identified in our case is a perforation of the atrial wall, fibrous pericardium, and pleura, extending to the RSPV, which caused low-grade bleeding into the pleural space (*Figure 4*).

This case report highlights the inherent limitations of conventional CT imaging in detecting low-volume, active intrathoracic haemorrhage. The CT scan failed to identify ongoing bleeding, which can be attributed to two primary factors: artefacts generated by the pacemaker lead and the low-flow nature of the haemorrhage. The latter is particularly challenging to detect without cardiac-gated acquisition protocols. The subtle, low-volume haemorrhage was likely below the detection threshold of standard CT imaging, underscoring the difficulties in visualizing minor intrathoracic bleeding events on non-ECG–synchronized scans. This diagnostic challenge underscores the potential for false-negative results in similar clinical scenarios. Moreover, this case emphasizes the critical importance of astute clinical judgment and meticulous evaluation of the temporal progression of symptoms in the context of post-interventional complications. The discrepancy between imaging findings and clinical presentation serves as a reminder that radiological investigations, while invaluable, should be interpreted in conjunction with a comprehensive clinical assessment to ensure accurate diagnosis and appropriate management of post-procedural complications.

## Conclusion

In conclusion, this case highlights a rare complication of pacemaker implantation: contralateral haemothorax due to RSPV laceration. It underscores the necessity for vigilant post-procedural monitoring and rapid intervention in high-risk patients. The challenges in detecting low-volume intrathoracic haemorrhage with standard imaging reinforce the importance of clinical judgment alongside radiological assessments.

## Supplementary Material

ytaf093_Supplementary_Data

## Data Availability

Data are available upon request from the corresponding author.

## Lead author biography

Simon Fitouchi is a cardiologist at the University Hospital of Strasbourg (France). He graduated from the University of Strasbourg in Cardiovascular Medicine in 2021 and is currently training in interventional rhythmology.

## Notes

**Consent:** The authors confirm that written consent for submission and publication of this case report including images and associated text has been obtained from the patient in line with the COPE guidelines.

**Funding:** This work was supported by GERCA (Groupe pour l'Enseignement, la prévention et la Recherche Cardiologique en Alsace).
