# Supplementary figures and images for: Right haemothorax secondary to pulmonary vein laceration following left-sided pacemaker implantation: a case report
Source: Eur Heart J Case Rep. 2025 Feb 25;9(3):ytaf093. doi: 10.1093/ehjcr/ytaf093 (PMC11911154; doi:10.1093/ehjcr/ytaf093)

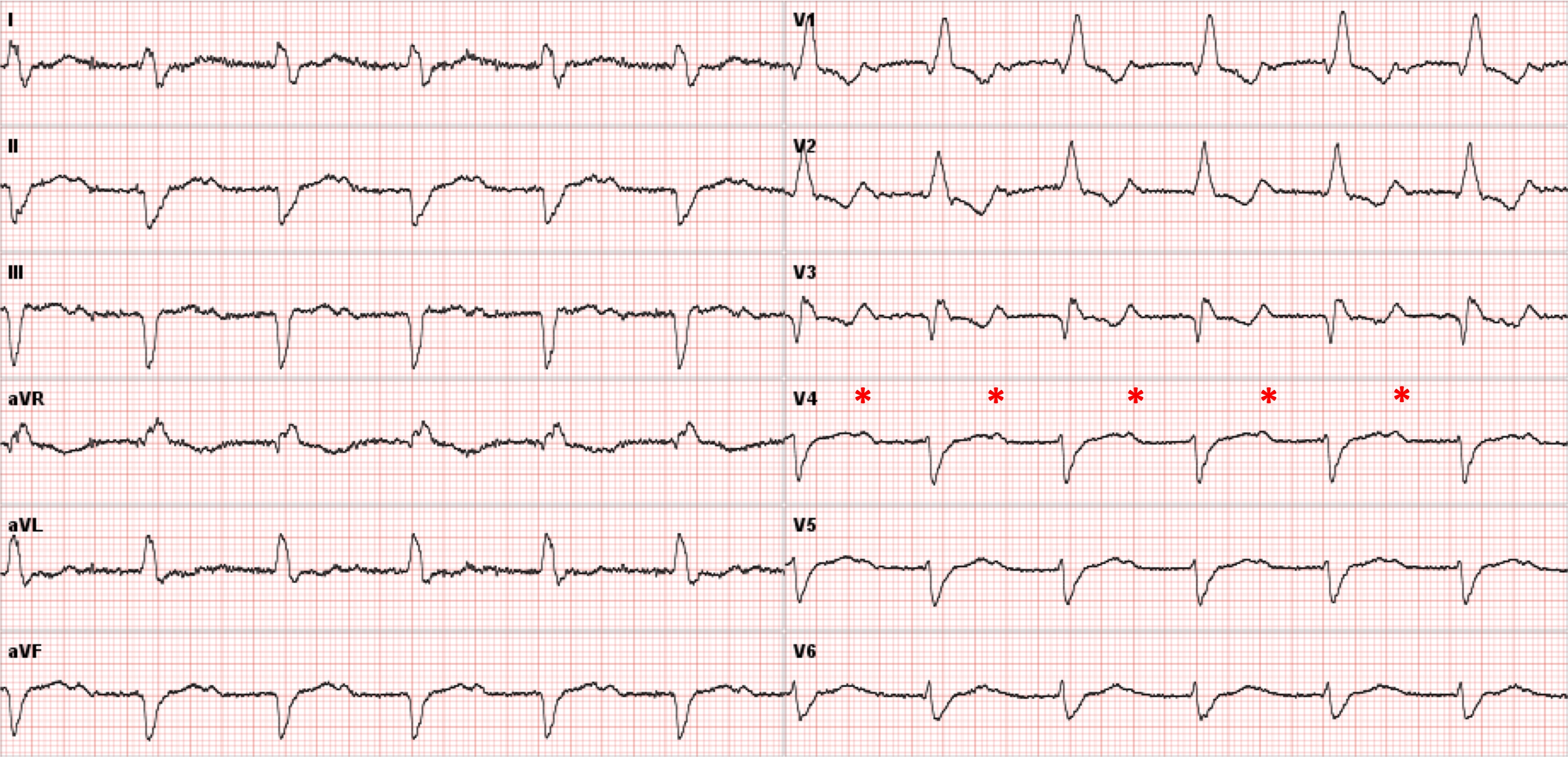

Supplement: ytaf093_Supplementary_Data [file ytaf093_supplementary_data.zip › Supplementary material, Figure S1.jpg]

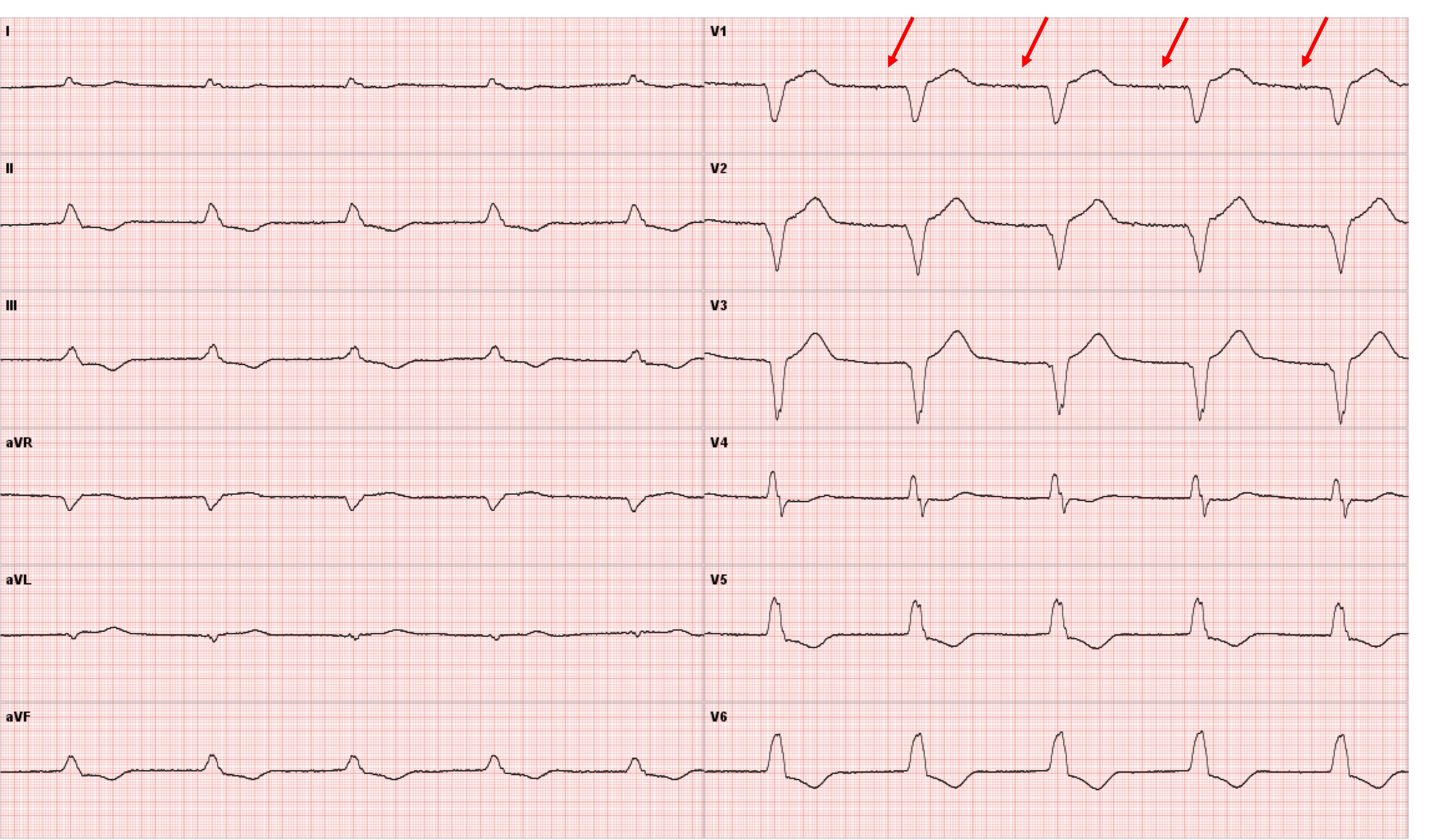

Supplement: ytaf093_Supplementary_Data [file ytaf093_supplementary_data.zip › Supplementary material, Figure S2.jpg]

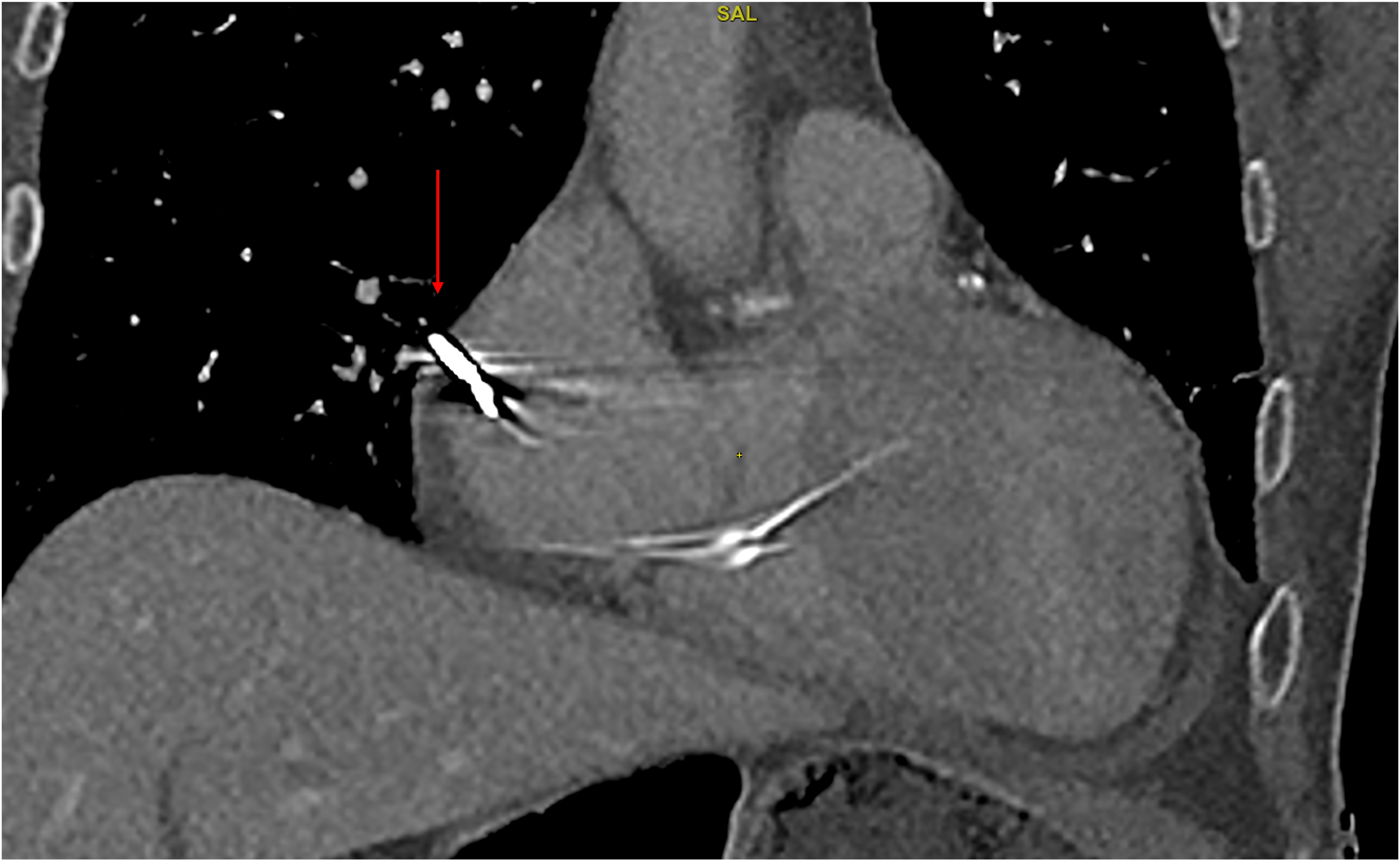

Supplement: ytaf093_Supplementary_Data [file ytaf093_supplementary_data.zip › Supplementary material, Figure S4.jpg]

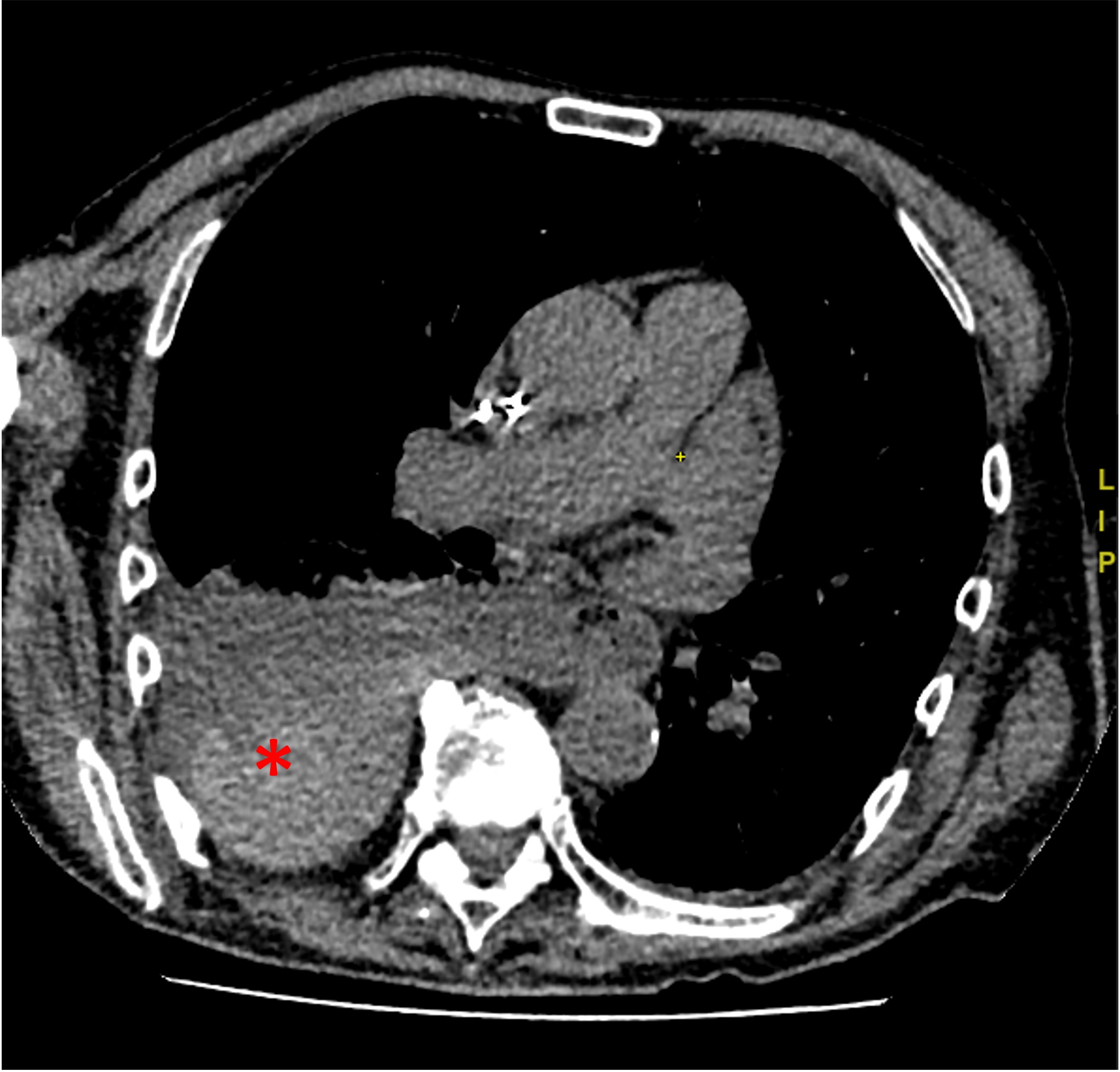

Supplement: ytaf093_Supplementary_Data [file ytaf093_supplementary_data.zip › Supplementary material, Figure S5.jpg]

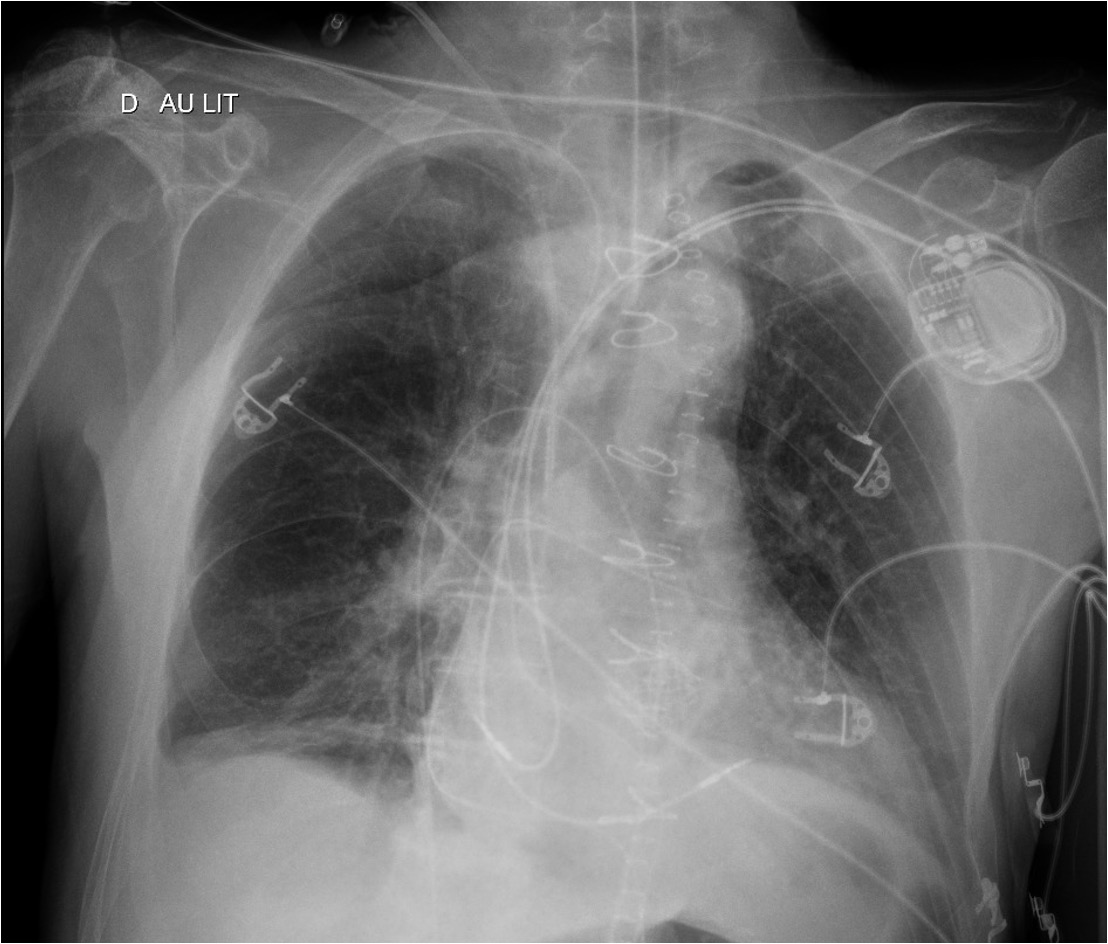

Supplement: ytaf093_Supplementary_Data [file ytaf093_supplementary_data.zip › Supplementary material, Figure S6.jpg]
